# Antivirulence Properties of a Low-Molecular-Weight Quaternized Chitosan Derivative against *Pseudomonas aeruginosa*

**DOI:** 10.3390/microorganisms9050912

**Published:** 2021-04-24

**Authors:** Giuseppantonio Maisetta, Anna Maria Piras, Vincenzo Motta, Simona Braccini, Diletta Mazzantini, Federica Chiellini, Ylenia Zambito, Semih Esin, Giovanna Batoni

**Affiliations:** 1 Department of Translational Research and New Technologies in Medicine and Surgery, University of Pisa, 56126 Pisa, Italy; vincenzo.motta@med.unipi.it (V.M.); diletta.mazzantini@med.unipi.it (D.M.); semih.esin@med.unipi.it (S.E.); giovanna.batoni@med.unipi.it (G.B.); 2 Department of Pharmacy, University of Pisa, 56126 Pisa, Italy; anna.piras@unipi.it (A.M.P.); ylenia.zambito@unipi.it (Y.Z.); 3 Department of Chemistry and Industrial Chemistry, University of Pisa, UdR INSTM PISA, 56124 Pisa, Italy; simona.braccini@phd.unipi.it (S.B.); federica.chiellini@unipi.it (F.C.); 4 Interdepartmental Research Centre “Nutraceuticals and Food for Health”, University of Pisa, 56100 Pisa, Italy

**Keywords:** chitosan, quaternized chitosan, *Pseudomonas aeruginosa*, pyocyanin, pyoverdin, proteases, LasA, motility, virulence factors, quorum sensing, biofilm, tobramycin

## Abstract

The co-occurrence of increasing rates of resistance to current antibiotics and the paucity of novel antibiotics pose major challenges for the treatment of bacterial infections. In this scenario, treatments targeting bacterial virulence have gained considerable interest as they are expected to exert a weaker selection for resistance than conventional antibiotics. In a previous study, we demonstrated that a low-molecular-weight quaternized chitosan derivative, named QAL, displays antibiofilm activity against the major pathogen *Pseudomonas aeruginosa* at subinhibitory concentrations. The aim of this study was to investigate whether QAL was able to inhibit the production of relevant virulence factors of *P. aeruginosa*. When tested in vitro at subinhibiting concentrations (0.31–0.62 mg/mL), QAL markedly reduced the production of pyocyanin, pyoverdin, proteases, and LasA, as well as inhibited the swarming motility of three out of four *P. aeruginosa* strains tested. Furthermore, quantitative reverse transcription PCR (qRT-PCR) analyses demonstrated that expression of *lasI* and *rhlI*, two QS-related genes, was highly downregulated in a representative *P. aeruginosa* strain. Confocal scanning laser microscopy analysis suggested that FITC-labelled QAL accumulates intracellularly following incubation with *P. aeruginosa*. In contrast, the reduced production of virulence factors was not evidenced when QAL was used as the main polymeric component of polyelectrolyte-based nanoparticles. Additionally, combination of sub-MIC concentrations of QAL and tobramycin significantly reduced biofilm formation of *P. aeruginosa*, likely due to a synergistic activity towards planktonic bacteria. Overall, the results obtained demonstrated an antivirulence activity of QAL, possibly due to polymer intracellular localization and QS-inhibition, and its ability to inhibit *P. aeruginosa* growth synergizing with tobramycin.

## 1. Introduction

*Pseudomonas aeruginosa* is an opportunistic Gram-negative bacterium and a leading cause of nosocomial infections, highly contributing to morbidity and mortality of patients with cystic fibrosis (CF) or with severe burns [1,2,3]. *P*. *aeruginosa* readily acquires antimicrobial resistance determinants, resulting in multidrug-resistant or even pan-drug-resistant strains. Hence, the World Health Organization placed *P. aeruginosa* on the top of the priority pathogen list for the development of new antibiotics [4].

The ability of *P. aeruginosa* to colonize different human body districts and to resist both the host immune response and antimicrobial therapy mainly relies on its capacity to finely modulate the expression of multiple virulence factors and to form biofilms [5,6]. In many pathogenic bacteria, virulence factors and biofilm formation are coregulated by cell-to-cell communication systems known as quorum-sensing (QS). QS allows both Gram-negative and Gram-positive bacteria to regulate gene expression in response to population density by releasing, sensing, and responding to signal molecules called autoinducers [7]. The QS of *P. aeruginosa* is comprised of at least three hierarchically integrated systems—Las, Rhl, and PQS—that control the expression of virulence factors (e.g., pyocyanin, pyoverdine, proteases, elastase, and motility) as well as genes involved in biofilm formation [8]. *P. aeruginosa* QS-deficient mutants display attenuated virulence in different animal models of infection, and for this reason QS is considered a good target for the development of *P. aeruginosa* antivirulence drugs [9,10].

Importantly, antivirulence drugs may have less or no impact on bacterial growth as compared with conventional antimicrobials, reducing the evolutionary drive of the pathogen to acquire resistance. Another advantage of antivirulence strategy is that only the pathogenic bacteria will be affected while the resident beneficial bacterial flora is kept intact. Finally, virulence-blocking agents are expected to act also on bacterial strains resistant to conventional antibiotics, circumventing the alarming spread of antimicrobial resistance.

Several synthetic and natural compounds have shown efficacy as antibiofilm and anti-QS agents [11,12]. Among them, Chitosan has recently attracted much interest as evidence is accumulating that the biopolymer extracted from different sources may interfere with QS and biofilm formation of bacterial pathogens [13,14]. Chitosan has long been known for its unique pharmacological and cosmetic properties such as antimicrobial activity, wound-healing effect, high biocompatibility, and low toxicity, but its characterization as an antivirulence compound is still at a very early stage. Physical-chemical features, such as deacetylation degree, molecular weight (MW), and poor water solubility at physiological pHs, are reported to greatly affect Chitosan’s antimicrobial activity, which is poor at pH values above six. To overcome the water solubility limitation and/or improve the biological activity of Chitosan, several N-modified and/or O-modified Chitosan derivatives have been described, including quaternized, phosphorylated, and acyl thiourea derivatives [15,16]. In particular, the insertion of a quaternary moiety allows permanent cationic charges to be obtained on the polysaccharide backbone and significantly improves properties such as water solubility, antimicrobial activity, and muco-adhesiveness [17].

In a previous study we demonstrated that a quaternary Chitosan-derivative (QAL)—characterized by low molecular weight, high degree of substitution (over 80%), and short chains containing two adjacent quaternary ammonium groups—possesses antibacterial, antibiofilm, and antiadhesive properties against *Staphylococcus epidermidis* and *P. aeruginosa*. [18]. The same derivative also demonstrated mucus-adhesive capability as well as wound-healing promotion, suggesting its possible use as a therapeutic agent in bacterial infections [19,20].

QAL and its derivatives have been deeply investigated and applied for drug delivery purposes either as jellying agents in their macromolecular forms or as nanoparticulate carriers. Additionally, depending on the investigated treatment, the macromolecular form and the nanoparticulate carriers showed diverse biopharmaceutical features, favouring the polymeric macromolecular form [21,22] or the nanoparticulate carrier [23,24], respectively. In view of maximizing QAL’s role as a functional agent/carrier in antivirulence applications, it appears mandatory to assess its antivirulence properties in the forms of both soluble macromolecular agent and colloidal nanosuspension.

Consequently, the main aim of this study was to investigate the antivirulence properties of the quaternary ammonium chitosan derivative QAL towards clinical strains of *P. aeruginosa*.

The results obtained demonstrated that polymeric QAL, but not QAL nanoparticles, is able to markedly reduce the production of relevant virulence factors of *P. aeruginosa*, possibly by accumulating intracellularly and inhibiting QS genes. Interestingly, QAL was also found to synergize with tobramycin, a drug largely employed in the treatment of *P. aeruginosa* infections, resulting in a significant reduction in the viable count of *P. aeruginosa* in planktonic, as well as in biofilm, modes of growth. Overall, our study provides supporting evidence that Chitosan’s derivatives may exert antivirulence properties and synergistic activity with conventional drugs, lengthening the list of the favourable pharmacological activities of these polymers.

## 2. Materials and Methods

### 2.1. Bacterial Strains

Three clinical strains of *P. aeruginosa* (W4, CVC02118, BAL0910), isolated at the microbiology laboratory of the University Hospital of Pisa, Italy, and the reference strain ATCC 27853 were used in the study. The main characteristics of the strains are summarized in Table 1. For the preparation of stock cultures, bacterial strains were grown in Luria Bertani broth (LB, Oxoid, Basingstoke, Hampshire, UK) until late-log phase, subdivided in aliquots, and kept frozen at −80 °C until use. Identification and susceptibility testing of *P. aeruginosa* strains were performed by MALDI-TOF (Bruker Daltonics, Bremen, Germany) and BD Phoenix susceptibility testing system (BD, Milan, Italy), respectively.

### 2.2. QAL and QAL-Based Nanoparticles

The low molecular weight *N,O*-[*N,N*-diethylaminomethyl(diethyldimethylene ammonium)_n_ methyl]chitosan (QAL) was freshly prepared according to previously described procedures [25]. As previously reported [18], QAL has a molecular weight of 163 kg/mol, deacetylation degree of 97%, and 80% of pendant quaternary chain (*n* = 2). The FITC-label QAL derivative (0.8% wt of FITC) was freshly prepared according to previously described procedures [21].

QAL-based nanoparticles (QAL-NP) were prepared by polyelectrolyte complexing with GENUVISCO type CSW-2 carrageenin (CG). CG was dissolved in water to a concentration of 0.5 mg/mL and 0.6 mL were added dropwise to 2.0 mL of QAL 1 mg/mL in water. QAL-NP were formed spontaneously after 1 h at room temperature.

For the microbiological analysis, the QAL-NP were sterilized in an autoclave at 121 °C for 20 min. Afterwards, the QAL-NP were concentrated by centrifugation and redispersed in 0.22 mm filtered water to a final concentration of 5 mg/mL. The protocol was performed under laminar flow cabinet (Steril-VHB Compact, Angelantoni Life Science s.r.l. (Massa Martana (PG), Italy)).

Nanoparticles were characterized in terms of size and zeta potential. Diameter distribution and zeta potential value were evaluated by Malvern Zetasizer Nano ZS (Malvern Panalytical Ltd., Malvern, UK).

### 2.3. Determination of Minimal Inhibitory Concentrations (MICs)

The susceptibility of *P. aeruginosa* strains to QAL was assessed in terms of MIC values according to the standard microdilution method (Clinical and Laboratory Standards Institute–CLSI, 2018) with some modifications. Briefly, bacteria were grown in LB broth (Oxoid), until exponential growth phase and diluted in the same medium to reach a final density of 5 × 10^6^ CFU/mL. A volume of 10 μL of the bacterial suspensions was added to 90 μL of LB in a 96-well plate in the absence (viability control) or in the presence of the compound at different concentrations. MIC values were defined as the lowest concentration of QAL resulting in the complete inhibition of visible growth after 24 h of incubation at 37 °C. Susceptibility of *P. aeruginosa* strains to QAL and tobramycin (Sigma Aldrich, Saint Louis, MO, USA) was also evaluated in 20% Tryptone Soy Broth (TSB, Oxoid) and MICs were determined as above.

### 2.4. Growth Curves

In order to assess whether QAL exerted a partial inhibitory effect at sub-MIC concentrations, growth curve analysis was performed. Overnight cultures of *P. aeruginosa* strains were diluted 1:100 in fresh LB broth both in the absence and presence of QAL at 0.31 and 0.62 mg/mL and incubated at 37 °C in an orbital shaker. The growth curves were then determined by measuring the OD at 570 nm at 1 h time intervals up to 10 h and at 24 h.

### 2.5. Assays for Evaluation of Virulence Factors in Culture Supernatants

*P. aeruginosa* strains were grown in LB broth for 18 h, then diluted 1:100 in LB and incubated in the absence or presence of QAL (0.155, 0.31, and 0.62 mg/mL) in static conditions at 37 °C for 48 h. Following incubation, the OD_600_ of the cultures was determined to estimate the bacterial density. After that, cultures were centrifuged twice at 2700× *g* for 15 min to assure removal of bacteria (Eppendorf centrifuge 5417R) at room temperature and culture supernatants were used for the quantification of virulence factors. The value obtained for each virulence factor was multiplied by the ratio OD_600_ of the control/OD_600_ of the sample, to normalize for small differences in the culture densities between the controls and QAL-exposed samples after 48 h of incubation. In some experiments, the bacterial load of treated and untreated samples was verified by plating and CFU count, resulting in no significant difference. The same experimental procedure was followed to evaluate the effects of QAL-NPs on the production of virulence factors by the *P. aeruginosa* W4 strain.

Pyocyanin pigment was extracted from cell-free supernatants by subsequent exposure to chloroform and 0.2 N hydrochloric acid (Sigma) and quantified at OD_520_, as previously described [26]. Pyoverdin was quantified by measuring the OD_400_ of supernatants. The total proteolytic activity of *P. aeruginosa* strains was determined using a modified skim milk assay [27]. Briefly, culture supernatants of *P. aeruginosa* strains (0.5 mL) were incubated with 0.5 mL skim milk (1.25%) (Fluka, Munich, Germany) at 37 °C for 30 min and turbidity was monitored at OD_600_ nm. The decrease in turbidity due to proteolytic activity was expressed as ΔA/min/mL. Secreted LasA of *P. aeruginosa* has a staphylolytic activity due its ability to hydrolyse pentaglycine bonds of *Staphylococcus aureus* peptidoglycan. LasA activity was assessed by monitoring the ability of supernatants from *P. aeruginosa,* exposed to QAL or not, to lyse boiled cells of *S. aureus* ATCC 33591 and to decrease the OD_600_. Enzyme activity was expressed as ΔA/min/mL [28].

### 2.6. Motility Assay

The ability of QAL to inhibit the QS-mediated swarming motility of *P. aeruginosa* was assessed by the soft agar method. Briefly, swarming soft medium (2.5 mg/mL NaCl; 30 mg/mL Na_2_HPO_4_; 15 mg/mL KH_2_PO_4_; 0.2% glucose, all from Sigma; 0.5% bacteriological agar; 0.5% casamino, all from Oxoid; and 1 mM MgSO_4_ Sigma) was poured into 60 mm plates in the absence and presence of 0.155, 0.31, and 0.62 mg/mL QAL and allowed to dry at room temperature for ~2 h prior to inoculation. Each plate was inoculated with 2.5 μL of a liquid culture grown for 18 h in LB broth. The plates were incubated face up at 37 °C for 24 h. The motility spread was measured with a micrometer and the results were expressed in mm; each experiment was carried out in triplicate.

### 2.7. Total RNA Isolation and Quantitative Reverse Transcription PCR (qRT-PCR) Analysis

The expression of the QS-related genes *lasI* and *rhlI* was determined by real-time quantitative PCR. To this aim, *P. aeruginosa* W4 was grown in LB broth for 18 h, then diluted 1:100 in LB and incubated with different doses of QAL (0.3 mg/mL, 0.6 mg/mL, control without QAL) at 37 °C for 48 h in static conditions. After washing of bacterial pellets with TE buffer, bacterial mRNA was extracted by Nucleospin RNA kit (Macherey-Nagel GmbH & Co., KG Düren, Germany) following the manufacturer’s instructions. The RNA purity and the yield of extraction were determined by measuring the OD_260_ and OD_280_ and determining A_260_:A_280_ ratio using an Eppendorf BioPhotometer D30 (Eppendorf AG, Hamburg, Germany). Then, 500 ng of the purified RNA was used as a template in one-step RT-PCR with TransScript One-Step gDNA Removal and cDNA Synthesis SuperMix (Transgenbiotech, Beijing, China). The cDNA was real-time amplified using Luna Universal qPCR Master Mix (New England Biolabs, Ipswich, MA, USA) on a CFX Connect Real-Time PCR Detection System (Bio-Rad, Feldkirchen, Germany). The expression of *lasI* and *rhlI* was analysed by the 2^−ΔΔCT^ method [29] using *rpoD* as a reference gene [30]. Three biological replicates and three technical replicates for each experiment were performed. The primers and the amplification conditions used in the present study are reported in Tables S1 and S2.

### 2.8. Flow Cytometry Analysis

*P. aeruginosa* W4 incubated for 48 h with QAL-FITC was analysed by flow cytometry. To this aim, following centrifugation at 2700× *g* for 15 min, the supernatant was discarded, and the bacterial pellet was resuspended in phosphate-buffered saline (PBS, Euroclone S.p.A, Pero, Milan). Following a wash (2700× *g*, 15 min), the pellet was resuspended in 4% paraformaldehyde (PFA, Sigma-Aldrich) and the bacteria were fixed for 30 min at room temperature. Following a centrifugation at 3000× *g* for 5 min, *P. aeruginosa* cells were resuspended in PBS at a density of approximately 1 × 10^7^ CFU/mL and at least 50,000 events were acquired ungated in a BD Accuri C6 flow cytometer (BD Biosciences, San Jose, CA, USA). BD Accuri C6 software (BD Biosciences) was used for computer-assisted analyses. For the analyses, bacteria cells were selected by a widely set gate on a two-parameter plot of side scatter versus forward-angle scatter; among these cells the percentage of the green fluorescent bacteria were calculated. Bacteria incubated for 48 h with FITC only or with unlabelled QAL represented negative controls.

### 2.9. Confocal Scanning Laser Microscopy (CLSM) Analysis

The cellular localization of QAL in *P. aeruginosa* W4 treated cells was evaluated by means of CLSM. To this aim, *P. aeruginosa* W4 was grown in LB broth for 18 h, then diluted 1:100 in LB and incubated with QAL-FITC at 0.62 mg/mL, or with FITC only (negative control) at 37 °C for 48 h in static conditions. Following the incubation, bacteria were washed once with PBS (2700× *g*, 15 min) and fixed with 4% PFA as described above. Following another wash with PBS, the samples were analysed with CLSM. A Nikon Eclipse TE2000 inverted microscope equipped with EZ-C1 confocal laser (Nikon, Tokyo, Japan) apparatus and a 100× oil-immersion objective were used to analyse samples. An argon-ion laser (488 nm emission) was used to excite FITC. Images were captured with Nikon EZ-C1 software with identical settings for each sample and Z-stack micrographs were obtained at intervals of 0.50 µm.

### 2.10. Biofilm Inhibition Assay

*P. aeruginosa* strains grown overnight in TSB at 37 °C were diluted 1:100 in 20% TSB. Bacterial suspensions were dispensed into flat-bottom polystyrene 96-well microplates (Corning Costar, Lowell, MA, USA) in the presence of sub-MIC concentrations of QAL (0.037, 0.075, and 0.15 mg/mL) and tobramycin (0.125 and 0.25 µg/mL) (Sigma), tested alone or in combination. Microplates were incubated statically at 37 °C for 24 h and biofilm biomass was estimated by crystal violet (CV) (Sigma-Aldrich) staining assay, as previously described [31].

### 2.11. Planktonic Growth Inhibition Assay

*P. aeruginosa* strains grown overnight in TSB broth at 37 °C were diluted 1:100 in 20% TSB. Bacterial suspensions were incubated in the presence of sub-MIC concentrations of QAL (0.037, 0.075, and 0.15 mg/mL) and tobramycin (0.125 and 0.25 µg/mL) (Sigma), tested alone or in combination, in polypropylene microtubes (1.5 mL) (Corning Costar, Lowell, MA, USA) at 37 °C for 24 h under agitation. The bacterial growth was evaluated by spectrophotometric measurement at OD_570_ (Multiscan FC, Thermo Fisher Scientific, Waltham, MA, USA).

### 2.12. Statistical Analysis

All the experiments were performed at least three times. Statistical analysis was carried out using GraphPad InStat software (GraphPad InStat Software, Inc., San Diego, CA, USA). Differences between mean values were evaluated with one-way analysis of variance (ANOVA) followed by Tukey–Kramer post-hoc test in the case of multiple comparisons. A *p*-value of < 0.05 was considered significant.

## 3. Results

### 3.1. Determination of MIC Values of QAL against Clinical Isolates of P. aeruginosa

The efficacy of QAL on planktonic cells of *P. aeruginosa* strains, isolated from different body districts, was investigated by determination of the MIC values. The least concentrations that inhibited the visible bacterial growth at 24 h of incubation ranged between 2.5 and 5 mg/mL (Table 1).

### 3.2. Effect of Subinhibiting Concentrations of QAL on Growth Curves

**Figure 1:**
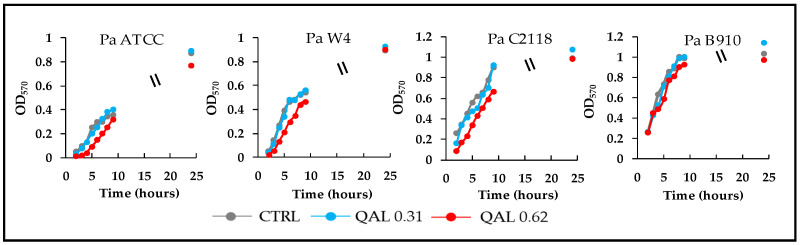
Growth curves of *P. aeruginosa* strains incubated in LB broth alone (CTRL, control) or in the presence of QAL 0.31 or 0.62 mg/mL. Values are the mean of two independent experiments.

In order to evaluate whether QAL at sub-MIC concentrations could affect the duplication rate of the *P. aeruginosa* strains under evaluation, growth curves were monitored for each strain in the presence of 0.31 and 0.62 mg/mL QAL. The results obtained demonstrated that at such concentrations QAL did not show any significant effect on the growth kinetics of all the four strains tested (Figure 1). Higher QAL concentrations inhibited the early growth of the strains although they were sub-MIC. On the basis of these results the concentrations of 0.15, 0.31, and 0.62 mg/mL QAL were selected for the subsequent antivirulence assays.

### 3.3. Effects of QAL on Virulence Factor Production

**Figure 2:**
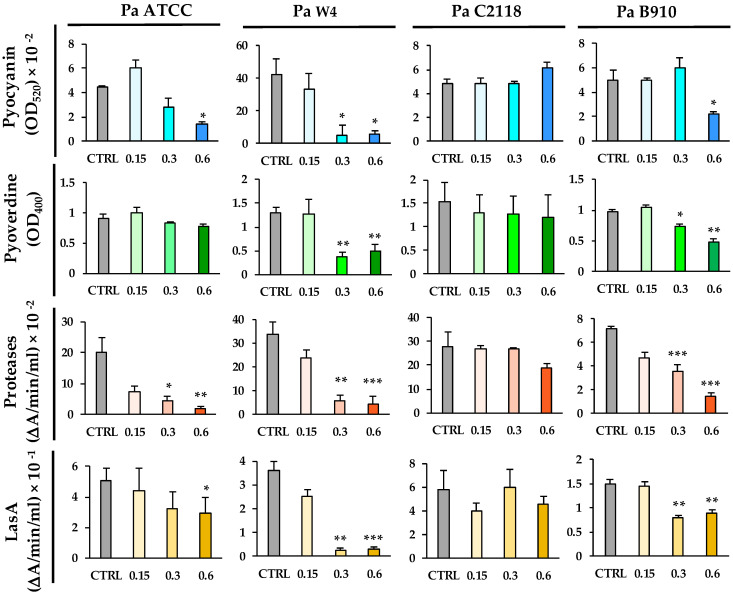
Effects of QAL at sub-MIC concentrations on the production of virulence factors by *P. aeruginosa* strains. Virulence factors were quantified in supernatant cultures after incubation of each bacterial strain in the presence or absence (CTRL) of QAL for 48 h in LB broth in static conditions. Each experiment was carried out at least three times. * *p* < 0.05; ** *p* < 0.01; *** *p* < 0.001.

Pyocyanin is a blue-redox pigment secreted by *P. aeruginosa*, cytotoxic for host cells through the generation of reactive oxygen species [32]. QAL caused a significant reduction in pyocyanin production in three out of four bacterial strains tested. In particular, pyocyanin levels in the supernatants of Pa ATCC, Pa W4, and Pa B910 were reduced by 60, 85, and 56%, respectively, when such bacterial strains were exposed to QAL 0.62 mg/mL (Figure 2). At the concentration of 0.31 mg/mL, QAL was still able to reduce the pyocyanin levels of Pa ATCC and Pa W4 by 38 and 77%, respectively (Figure 2). In contrast, QAL did not exert any evident effect on pyocyanin production by the Pa C2118 strain when tested up to the concentration of 0.62 mg/mL (Figure 2).

Pyoverdine is a fluorescent siderophore of *P. aeruginosa* which plays a crucial role in the host-bacterium interaction [33]. At 0.62 mg/mL, QAL inhibited pyoverdine production in Pa W4 and Pa B910 by 62 and 50%, respectively (Figure 2). At the same concentration, it did not significantly reduce pyoverdine production in Pa ATCC or Pa C2118 (Figure 2).

*P. aeruginosa* produces and secretes a number of proteases—such as LasA, elastase B (LasB), protease IV, and alkaline protease—which are considered important virulence factors as they damage host tissues and interfere with host antibacterial defence mechanisms [34]. The total proteolytic activity of Pa ATCC, Pa W4, and Pa B910 was markedly reduced (up to 90%) after exposure to QAL at the concentrations of both 0.62 and 0.31 mg/mL (Figure 2). The proteolytic activity of Pa C2118 was slightly reduced in the presence of QAL 0.62 mg/mL, but the difference did not reach statistical significance (Figure 2).

LasA, a zinc-dependent metalloprotease secreted by *P. aeruginosa* and endowed with staphylolytic activity, enhances the elastolytic activity of LasB and causes cellular damage in vivo [35]. LasA, evaluated in terms of staphylolytic activity, was significantly decreased when Pa ATCC, Pa W4, and Pa B910 were exposed to 0.62 and 0.31 mg/mL QAL (Figure 2). No significant reduction in LasA activity was observed after exposing Pa C2118 to QAL at the same concentrations (Figure 2).

### 3.4. Inhibition of Swarming Motility by QAL

**Figure 3:**
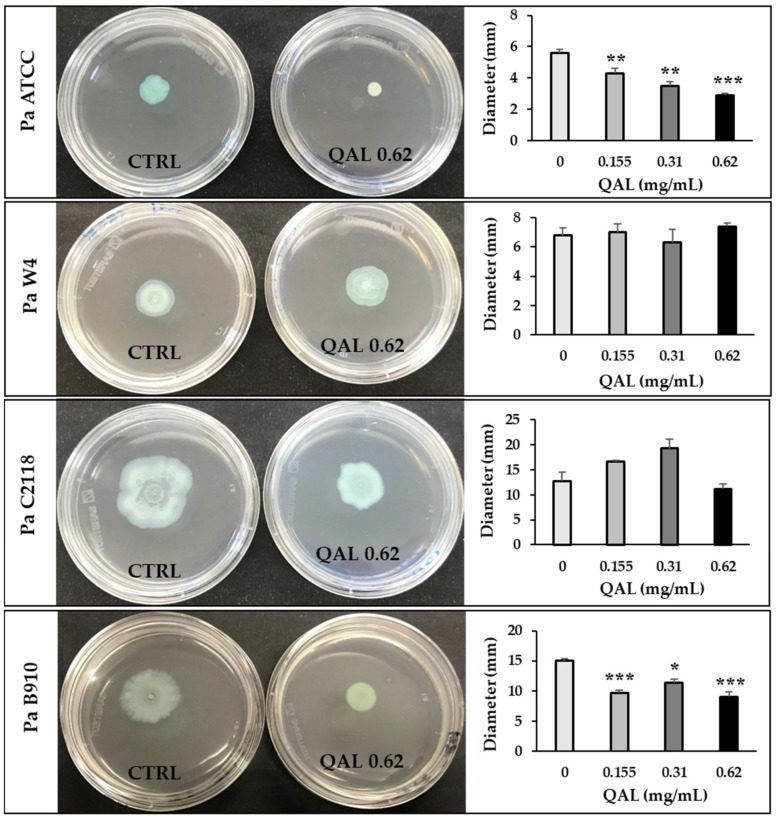
Effect of QAL at sub-MIC levels on swarming motility of *P. aeruginosa* strains. Swarming motility was evaluated by measuring the diameter of the swarming area (mm) on agar soft medium after incubation at 37 °C for 24 h. Each experiment was carried out three times. * *p* < 0.05; ** *p* < 0.01; *** *p* < 0.001. Representative swarm plate images taken 24 h after inoculation are also shown for each strain. Plates without QAL (CTRL, on the left) and plates containing QAL 0.62 mg/mL (on the right) are shown.

Swarming motility is a flagellar surface-associated motility that allows bacteria to rapidly colonise a surface, leading to initiation of biofilm formation [36]. To explore the effect of QAL on this motility type in *P. aeruginosa*, swarming assays were carried out. As shown in Figure 3, QAL significantly attenuated the swarming motility of Pa ATCC and Pa B910 strains at the lowest concentration of 0.155 mg/mL. QAL at the concentration of 0.62 mg/mL caused a 52 and 40% reduction in the swarming motility of Pa ATCC and Pa B910 strains, respectively (Figure 3). In contrast, no statistically significant reduction in swarming motility was observed for Pa W4 and Pa C2118 strains (Figure 3).

### 3.5. Effects of QAL on lasI and rhlI Gene Expression

**Figure 4:**
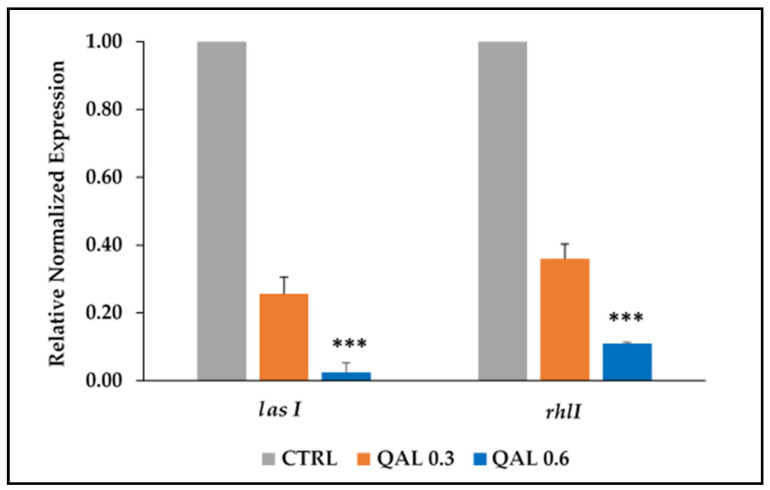
Relative expression of the QS-associated genes (*rhlI* and *lasI*) evaluated by qRT-PCR in *P. aeruginosa* cells untreated (CTRL) or incubated with QAL (0.31 and 0.62 mg/mL) for 48 h. The levels of QS gene expression were normalized to those of the house-keeping *rpoD* gene and then compared with the level of expression of the control. Results are expressed as the mean and standard error of the mean from three independent experiments. Statistically significant differences (** *p* < 0.01; *** *p* < 0.001) between the test compound and the corresponding controls are indicated.

Since the virulence factors of *P. aeruginosa* tested in this study are under the control of the QS system, we assessed the ability of QAL to inhibit the quorum-sensing-associated genes *lasI* and *rhlI* of the PaW4 strain, chosen as a representative strain. The results of qRT-PCR indicated that both genes were downregulated by the treatment of QAL in a dose-dependent manner (Figure 4). In particular, the higher concentration (0.62 mg/mL) of QAL caused a 42.09 (±5.87)-fold downregulation of *lasI* and a 9.50 (±1.92)-fold downregulation of *rhlI* transcript levels, whereas the lower concentration of QAL (0.31 mg/mL) reduced *lasI* and *rhlI* expression levels 4 (±0.67)-fold and 2.79 (±0.16)-fold, respectively (Figure 4).

### 3.6. Cellular Localization of QAL

To provide insight into the possible antivirulence mechanism of QAL, we sought to investigate the cellular localization of QAL in *P. aeruginosa*-treated cells. To this aim, Pa W4 strain was incubated for 48 h with 0.62 mg/mL FITC-labelled QAL in static conditions at 37 °C. After verifying that labelling of QAL with FITC did not alter the ability of the polymer to inhibit virulence factors production, we proceeded to analyse *P. aeruginosa* cells exposed to QAL-FITC by flow cytometer and confocal microscopy.

**Figure 5:**
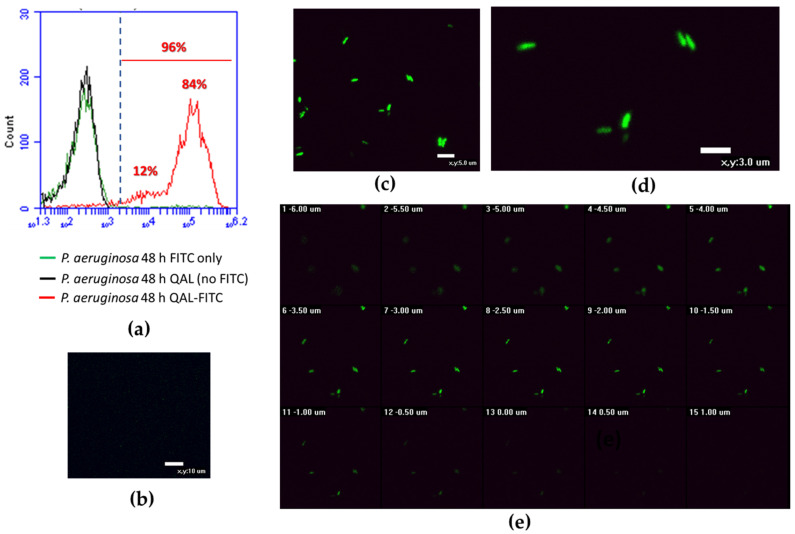
Flow cytometric analysis and CLSM images of *P. aeruginosa* cells incubated for 48 h with QAL-FITC at 0.62 mg/mL; (**a**) Cytofluorimetric analysis of QAL-FITC-treated *P. aeruginosa*; (**b**) CLSM image of *P. aeruginosa* treated with FITC only (negative control); (**c**,**d**) different fields of view of *P. aeruginosa* treated with QAL-FITC showing typical cytoplasmic granular fluorescent staining; (**e**) confocal Z-stack images of QAL-FITC-treated bacterial cells. A representative experiment is depicted out of two performed with similar results.

Cytofluorimetric analysis of QAL-FITC-treated Pa W4 revealed that up to 96% of bacteria were fluorescent as compared with the negative controls (*P. aeruginosa* incubated with unlabelled QAL and *P. aeruginosa* incubated with FITC only) (Figure 5a). On the other hand, CLSM analysis of *P. aeruginosa* treated with FITC-labelled QAL showed typical cytoplasmic granular fluorescent staining (Figure 5c,d), while no fluorescent signal was evident in bacteria treated only with FITC (Figure 5b). Furthermore, confocal Z-stack images of QAL-FITC-treated bacterial cells demonstrated an increasing fluorescent signal, proceeding from the outside to the inside of the cells, and a decreasing fluorescence intensity from the inside to the outside of the cells (Figure 5e), suggesting an intracellular localization of the polymer.

### 3.7. Preparation, Characterization, and Antivirulence Activity of QAL Nanoparticles (QAL-NP)

QAL-NP were obtained as the polyelectrolyte complex between the polycation QAL and the polyanion CG. The latter used as crosslinking agent. QAL-NP have a narrow diameter distribution with an average diameter of 236 ± 5.3nm, polydispersity index (PI) of 0.239 ± 0.01 and zeta potential value of +37.2 ± 8.6 mV (pH 7). The sterilization did not affect the stability of QAL-NP, displaying 236 ± 5.5 nm and 0.229 ± 0.010 as average diameter and PI, respectively. QAL-NP were tested for their ability to inhibit virulence factor production by *P. aeruginosa*. The Pa W4 strain was arbitrarily selected to this aim, as it was the most susceptible strain to the antivirulence activity of QAL. Unexpectedly, in contrast to what was observed with polymeric QAL, QAL-NP did not significantly reduce virulence factor production by the Pa W4 strain (Figure S1).

### 3.8. Synergistic Effect of QAL in Combination with Tobramycin against P. aeruginosa Strains in Biofilm and Planktonic Modes of Growth

**Figure 6:**
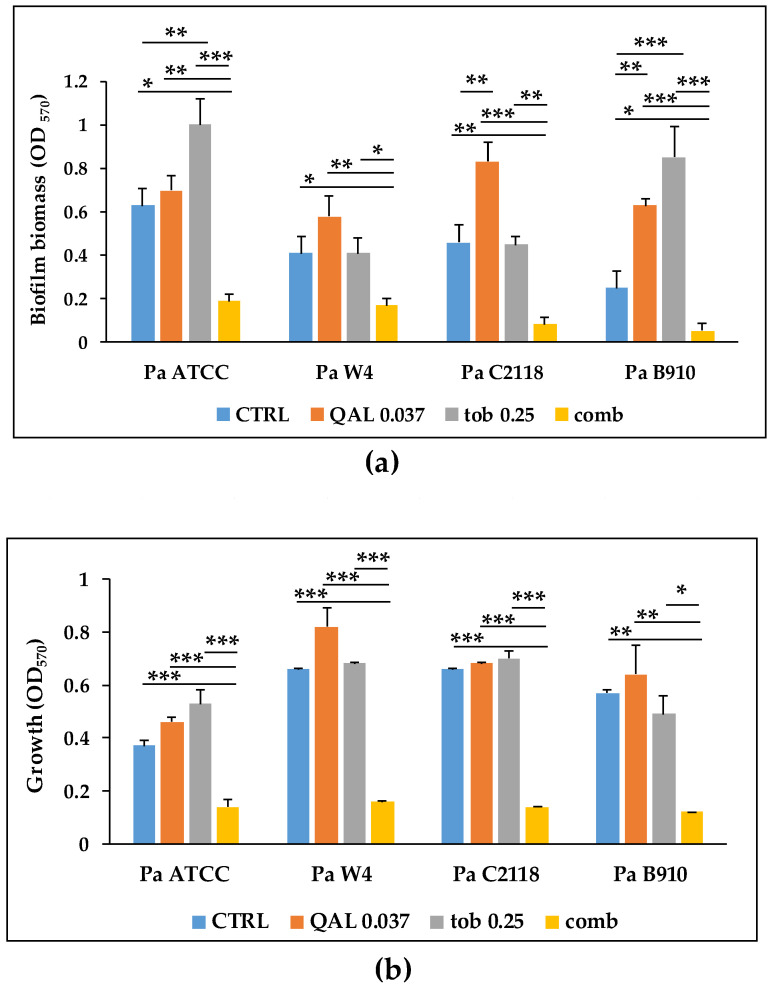
Effect of subinhibiting concentrations of QAL used alone and in combination (comb) with tobramycin (tob) on (**a**) biofilm formation and (**b**) planktonic growth of *P. aeruginosa* strains. CTRL, control. Data represent the average of two technical replicates from three independent experiments and error bars indicate standard error of the mean. Asterisks represent statistical significance of the comparisons between each sample with the corresponding combination. * *p* < 0.05; ** *p* < 0.01; *** *p* < 0.001.

In our previous study, QAL exhibited antibiofilm activity against *P. aeruginosa* [18]. In order to evaluate if QAL could synergize with tobramycin, an antibiotic widely used for the therapy of *P. aeruginosa* infections, we first identified the subinhibiting (sub-MIC) concentrations of both compounds in 20% TSB, the medium used to promote biofilm formation of the *P. aeruginosa* strains under evaluation (Table S3). Such concentrations were used alone and in combination in biofilm inhibition assays. Among the combinations tested, QAL at 0.037 mg/mL in combination with tobramycin at 0.25 µg/mL showed a marked synergistic antibiofilm effect against all four *P. aeruginosa* strains tested (Figure 6a). In particular, this combination caused a reduction of approximately 90% in biofilm formation compared with the control for all bacterial strains tested (Figure 6a). Similar synergistic effect was also observed when QAL and tobramycin were combined against *P. aeruginosa* strains grown in planktonic form (Figure 6b).

## 4. Discussion

*P. aeruginosa* is an extremely versatile bacterium with the ability to colonize a number of different environments and host tissues, resulting in a wide range of infection types [37]. This extraordinary ability is mostly ascribed to the production of many virulence factors that allow the bacterium to colonize, invade, and persist in host tissues; find nutrients; evade host immunity; and cause inflammation and tissue damage [38]. For instance, similarly to most bacterial pathogens, *P. aeruginosa* needs substantial amounts of iron to infect the host and multiply within tissues and body fluids. To retrieve iron, it produces two siderophores, pyoverdine and pyochelin, capable of chelating ferric ions (Fe^3+^) [39]. *P. aeruginosa* mutants deficient of pyoverdine were shown to be less virulent than the wild type strain in the burned-mouse model [40]. Furthermore, a strong correlation has been reported between pyoverdine production by *P. aeruginosa* strains isolated from CF patients and virulence in the *Caenorhabditis elegans* model, with pyoverdine inhibitors significantly improving survival of the worms [41].

In the era of antimicrobial resistance, antivirulence strategies have been endorsed as an attractive approach to control bacterial infections. Instead of imposing a selective pressure on bacterial growth, many virulence-suppressing compounds can attenuate the production of virulence factors and QS-based bacterial communication, disarming the pathogens and leading to reduced pathogenicity [10,12,42].

While the pharmacological features of Chitosan (e.g., biocompatibility, biodegradability, antimicrobial, and wound-healing activity) are well known—promoting its application in various fields [43]—much less investigated are the antivirulence properties of the polymer. In a previous study, we evaluated the antibacterial and antibiofilm properties of quaternized Chitosan-derivatives designed to overcome the water solubility limitation of Chitosan and improve its intrinsic antimicrobial activity [18]. Among them, the low-molecular-weight derivative, QAL, exhibited poor antibacterial activity against *P. aeruginosa*, but inhibited biofilm formation of the *P. aeruginosa* ATCC 27853 strain at sub-MIC concentrations [18]. Since both biofilm formation and synthesis of many virulence factors of *P. aeruginosa* are coregulated under the control of QS-signals, we hypothesized that QAL could also reduce the production of QS-related virulence factors such as pyoverdin, pyocyanin, proteases, and LasA.

In order to test QAL as an antivirulence agent, we first evaluated the antimicrobial properties of the compound in standard MIC assays to identify sub-MIC concentrations to use in the antivirulence assays. In agreement with our previous study [18], when tested in LB medium QAL inhibited the growth of different *P. aeruginosa* strains, but only at relatively high concentrations (5–2.5 mg/mL). This finding supports other studies in the literature pointing to a low activity of quaternized polymers against Gram-negative bacteria [44,45].

We next proceeded to assess the antivirulence properties of QAL against the *P. aeruginosa* reference strain ATCC 27853, as well as three clinical strains of *P. aeruginosa*—named Pa W4, Pa B910, and Pa C2118—isolated from wound, sputum, and CVC, respectively. In order to exclude that the eventual antivirulence effect of QAL was due to a growth-inhibiting effect of the compound at early incubation times, we first identified the maximum sub-MIC concentrations of QAL that could be used without effecting the growth kinetics of the four tested *P. aeruginosa* strains. When tested at such sub-MIC concentrations, QAL exhibited marked antivirulence effects towards the Pa ATCC, Pa W4, and Pa B910 strains, whereas the production of none of the virulence factors analysed was affected by QAL in the Pa C2118 strain at the tested concentrations. Included in the virulence factors inhibited by QAL was pyocyanin, a redox-active molecule which causes oxidative stress in host-cells and is reported to be a terminal-signalling factor in the QS network of *P. aeruginosa* [46]. At 0.62 mg/mL, QAL reduced the production of pyocyanin by 2.27- to 6.8-fold, depending on the strain. Of note, the levels of pyocyanin produced by PaW4 were markedly higher than those produced by the other *P. aeruginosa* strains were. Such an observation is in agreement with previous studies in the literature reporting differences higher than 10-fold in pyocyanin levels among different *P. aeruginosa* clinical strains isolated from sputum or chronic wounds [47,48]. Interestingly, it has been also reported that pyocyanin plays an important role in *P. aeruginosa* infections of chronic wounds [48]. In particular, pyocyanin was detected at high levels in the wounds of burn patients infected with *P. aeruginosa* and such levels were sufficient to induce normal human skin fibroblasts to become prematurely senescent, compromising tissue regeneration and wound repair [49]. It is tempting to speculate that the high levels of pyocyanin produced by the Pa W4 strain may represent an adaptation of the strain to the environment of the chronic wound from where it was isolated.

Similarly, QAL reduced the production of the siderophore pyoverdine, regulated by both the Las and PQS systems, in the PaW4 and Pa B910 strains.

*P. aeruginosa* proteases and LasA are regulated by the Las system and have a role in bacterial colonization and host cell damage [50]. The production of proteases and LasA appeared to be highly sensitive to QAL treatment, with both virulence factors being markedly reduced in the presence of 0.31 mg/mL QAL. Motility, regulated by the PQS system, is also considered a virulence property of *P. aeruginosa* [51]. The flagella-mediated surface movements such as swimming and swarming play a determining role in the bacterial initial attachment and colonization processes [52]. QAL decreased swarming motility by about 50% in the Pa ATCC and Pa B910 strains. Among the strains tested, only Pa C2118, isolated by a central venous catheter, proved to be quite insensitive to the antivirulence activity of QAL. Variability in the sensitivity to antivirulence and/or anti-QS agents among clinical *P. aeruginosa* isolates has been previously reported [53,54] and needs careful consideration when planning the use of such agents for therapeutic purposes. In a recent study, ciprofloxacin and a number of widely used disinfectants were found to reduce, or, to induce higher expression levels of QS-related genes in different *P. aeruginosa* strains [55]. These observations warrant future studies aimed at testing antivirulence agents, used alone or in combination with each other, against large panels of clinical isolates to fully explore the therapeutic potential of virulence-suppressing compounds.

A few studies have previously tested the antivirulence properties of native Chitosan. For instance, Chitosan purified from the fungus *Aspergillus flavus* or extracted from crab shells was found to inhibit the production of some QS-dependent virulence factors such as pyocyanin and proteases, as well as the expression of *lasR* and *rhl*R genes of *P. aeruginosa* [13,14]. Furthermore, low-molecular-weight variants of Chitosan obtained by irradiation of Chitosan extracted from crab shells were also found to reduce the synthesis of the virulence extracellular compound 4-hydroxy-2-alkylquinoline (HAQ), which has a role in QS of *P. aeruginosa* [56]. The results obtained in the present study indicate that the chemical modifications, introduced to obtain QAL, did not affect the antivirulence properties of Chitosan, opening new opportunities to further optimize the biological properties of such polymers via derivatization.

Many of the virulence factors secreted by *P. aeruginosa* are positively regulated by the QS systems of the bacterium [57,58,59]. In different animal models of infections such as acute or chronic lung infections [60,61], or burn wound infections [62], QS-deficient mutants of *P. aeruginosa* display reduced virulence, as compared with the parental QS-competent strains, suggesting that QS may provide the bacterium with a competitive advantage in the pathogenic interaction with the host. Thus, to ascertain the ability of QAL to inhibit the QS system at the transcriptional level and to support the inhibition of virulence factors observed at the phenotypic level, we evaluated the levels of expression of two QS-associated genes in the clinical isolate PaW4 exposed to subinhibitory concentrations of QAL. QAL reduced the expression of two of the main *P. aeruginosa* QS-associated genes, *lasI* and *rhlI*, which encode for autoinducer synthetases and control the production of various virulence factors in *P. aeruginosa* [63]. The downregulating effect of QAL was particularly evident on the *lasI* gene (about 50-fold) and, to a lesser extent, on the *rhlI* gene (about 9-fold). The QS systems in *P. aeruginosa* are strictly interconnected and the Las system is at the top of the signalling hierarchy [58,59], which possibly explains the differential activity of QAL observed on the two genes. The results obtained at the transcriptional level suggest that the inhibition of virulence factors observed at the phenotypic level might be due, at least partially, to an inhibition of QS-sensing signals.

The molecular mechanism(s) by which QAL can decrease the production of virulence factors and/or the transcription rate of QS-genes in *P. aeruginosa* is not known. However, a large number of reports discuss Chitosan’s antimicrobial mechanisms [64]. The first of these mechanisms is attributed to the polycationic nature of Chitosan, which promotes the polymer’s interaction with the negatively charged phospholipids of microbial cell surfaces, leading to the leakage of intracellular content and cell death [65]. Another proposed mechanism involves the binding of amino groups on Chitosan with the negatively charged phosphate groups of DNA by electrostatic interactions, resulting in the inhibition of transcription [66]. Recent molecular docking studies of Chitosan–DNA interaction confirmed the ability of Chitosan to bind to the major groove of DNA [67].

Thus, if Chitosan can pass through the cell membrane of Gram-negative bacteria, it could interact with DNA, interfering with the expression of genes encoding for virulence factors and/or related to the QS. In this study, confocal microscopy analysis suggested that FITC-labelled QAL, tested at the same subinhibiting concentrations used in the antivirulence assays, localize intracellularly in the Pa W4 strain. This finding is in agreement with other sporadic studies, in which FITC–labelled Chitosan oligomers or Chitosan nanoparticles were found to localize intracellularly when incubated with *Escherichia coli* [66,68]. Although more evidence is needed to prove the ability of Chitosan to permeate the bacterial cell membrane, our observation strengthens the hypothesis the sublethal concentrations of low-molecular-weight Chitosan derivatives may localize intracellularly and possibly interfere with DNA-expression processes.

To the perspective of using QAL as a multifunctional vehicle for antimicrobial drugs, QAL-based nanoparticles were prepared and tested for their ability to inhibit the synthesis of virulence factors of *P. aeruginosa.* Despite the finding that strain Pa W4 was particularly sensitive to QAL, QAL-NP did not provide any antivirulence activity against the same strain. The reasons for such inactivity of QAL-NPs are not known. The complexing of QAL with the carrageenan, and the formation of relevant nanoparticles, mainly constrains QAL mobility, which remains physically crosslinked into the polyelectrolyte complex (PEC) network. Since a charge unbalance was used for the PEC preparation and no chemical reaction occurs, the chemistry of the NP surface reflects that of plain QAL. QAL exposure on the QAL-NP surface was further demonstrated by the high positive zeta potential value. However, the 200 nm scale hydrodynamic diameter of QAL-NP is one hundred magnitude higher than that of QAL in its macromolecular form [21], providing a greater steric hindrance to molecular diffusion and to cellular internalization. Additionally, we can speculate that QAL constraint into QAL-NP recall the differences in molecular weight and charge density between low- and high-molecular-weight derivatives, which affected and favour the antibiofilm activity of the low-molecular-weight derivatives [18]. Overall, the diverse features of QAL and QAL-NP have a role in influencing the interactions of the two preparations with negatively charged bacterial surfaces.

An ineffective interaction of QAL-NPs with the bacterial cytoplasmic membrane might translate into a reduced ability of the nanoparticles to accumulate intracellularly, and then to inhibit the synthesis of virulence factors of *P. aeruginosa*.

Numerous antivirulence strategies are in the discovery or preclinical development phase [11,69]. Since inhibitors of virulence factors can only disarm the pathogens, but do not ensure eradication of pathogens, it is widely proposed that, once approved for clinical use, the combination of antivirulence agents with antibiotics could be an interesting approach. The killing/inhibiting effect of the antibiotic combined with the antivirulence/antibiofilm activity of the virulence-suppressing drug could produce a synergistic effect allowing for a decrease in therapeutic doses of both, thus helping to face antimicrobial resistance and toxicity-related issues. In this context, a number of studies have examined combinations of antibiotics and antivirulence compounds targeting various virulence factors including QS, iron uptake, and biofilm formation in *P*. *aeruginosa* [70,71,72].

In this study, the antibiofilm activity of QAL was tested in combination with tobramycin, an antibiotic commonly used for the therapy of infections sustained by *P. aeruginosa* [73,74]. Interestingly, QAL and tobramycin at concentrations below their MIC values markedly inhibited (90%) the biofilm formation of all *P. aeruginosa* strains tested. At the same concentrations, QAL in combination with tobramycin also reduced the planktonic growth of *P. aeruginosa* strains, suggesting that the antibiofilm effect was due to a synergistic effect of these compounds at the planktonic level. Tobramycin is an aminoglycoside which prevents protein biosynthesis by causing translational errors and by inhibiting translocation [75]. Based on this mechanism of action, we speculate that QAL at subinhibiting concentrations may transiently destabilize the bacterial membrane, facilitating the diffusion of tobramycin across the outer membrane or increasing the active uptake across the cytoplasmic membrane.

## 5. Conclusions

In this study, we demonstrated that the macromolecular form of the quaternized Chitosan-derivative QAL only, and not its nanoparticulate form QAL-NP, reduces the production of relevant virulence factors in *P. aeruginosa.* The antivirulence activity of QAL was evidenced at concentrations unable to affect the bacterial growth. Moreover, QAL inhibits QS-related genes of *P. aeruginosa* and it likely localizes intracellularly. We also showed that QAL synergizes with tobramycin in inhibiting both planktonic and biofilm growth of *P. aeruginosa*. Overall, such observations add antivirulence activity to the numerous pharmacological properties of Chitosan or its quaternized derivatives and point to QAL as a valuable multifunctional polymer to develop novel treatment strategies against *P. aeruginosa* infections.

## Figures and Tables

**Table 1:** MIC values of QAL towards *P. aeruginosa* strains.

Strain	Abbreviation	Isolation Site	Resistance Profile	MIC (mg/mL)
*P. aeruginosa* ATCC 27853	Pa ATCC	Blood	none	5
*P. aeruginosa* W4	PaW4	Diabetic wound	none	5
*P. aeruginosa* CVC02118	Pa C2118	Central venous catheter	CAZ, PIP, TZP	2.5
*P. aeruginosa* BAL0910	Pa B910	BAL	CIP, LVX	5

CAZ, ceftazidime; CIP, ciprofloxacin; LVX, levofloxacin; PIP, piperacillin; TZP, tazobactam/piperacillin; BAL, bronchoalveolar lavage.

## Data Availability

Data are available upon request to the corresponding author.

## Supplementary Materials

The following are available online at https://www.mdpi.com/article/10.3390/microorganisms9050912/s1, Table S1: Primers used in the study, Table S2: Amplification conditions, Table S3: MIC values of QAL towards *P. aeruginosa* strains in 20% TSB, Figure S1: Assays to evaluate antivirulence activity of QAL-NPs against the PaW4 strain.
