# Supplementary material for: Antivirulence Properties of a Low-Molecular-Weight Quaternized Chitosan Derivative against Pseudomonas aeruginosa
Source: Microorganisms. 2021 Apr 24;9(5):912. doi: 10.3390/microorganisms9050912 (PMC8145479; doi:10.3390/microorganisms9050912)
Supplement: Supplementary file 1 [file microorganisms-09-00912-s001.zip › microorganisms-1191356-supplementary.pdf]

Supplementary materials

Anti-virulence properties of a low-molecular quaternized chitosan derivative against *Pseudomonas aeruginosa*

Giuseppantonio Maisetta [1]*, Anna Maria Piras[2], Vincenzo Motta[1], Simona Braccini[3], Diletta Mazzantini[1], Federica Chiellini[3], Ylenia Zambito[2,4], Semih Esin[1], and Giovanna Batoni[1]

Table S1: Primers used in the study

Oligo Name	Sequence (5'-3')	Reference
LasI_For	CGCACATCTGGGAACTCA	[30]
LasI_Rev	CGGCACGGATCATCATCT	
RhlI_For	GTAGCGGGTTTGCGGATG	[30]
RhlI_Rev	CGGCATCAGGTCTTCATCG	
RpoD_For	CGAACTGCTTGCCGACTT	[30]
RpoD_Rev	GCGAGAGCCTCAAGGAATC	

Table S2: Amplification conditions

RhlI		LasI	
95 °C 2'		95 °C 2'	
95°C 15''	x 40	95°C 15''	x 40
58 °C 30''		56 °C 30''	
72 °C 5''		72 °C 5''	

Table S3. MIC values of QAL towards *P. aeruginosa* strains in TSB 20%

Strain	Abbreviation	QAL (mg/ml)	tob (μg/ml)
P. aeruginosa ATCC 27853	Pa ATCC	0.62	1
P. aeruginosa W4	Pa W4	0.62	1
P. aeruginosa CVC02118	Pa C2118	0.31	1
P. aeruginosa BAL0910	Pa B910	0.62	1

tob: tobramicyn

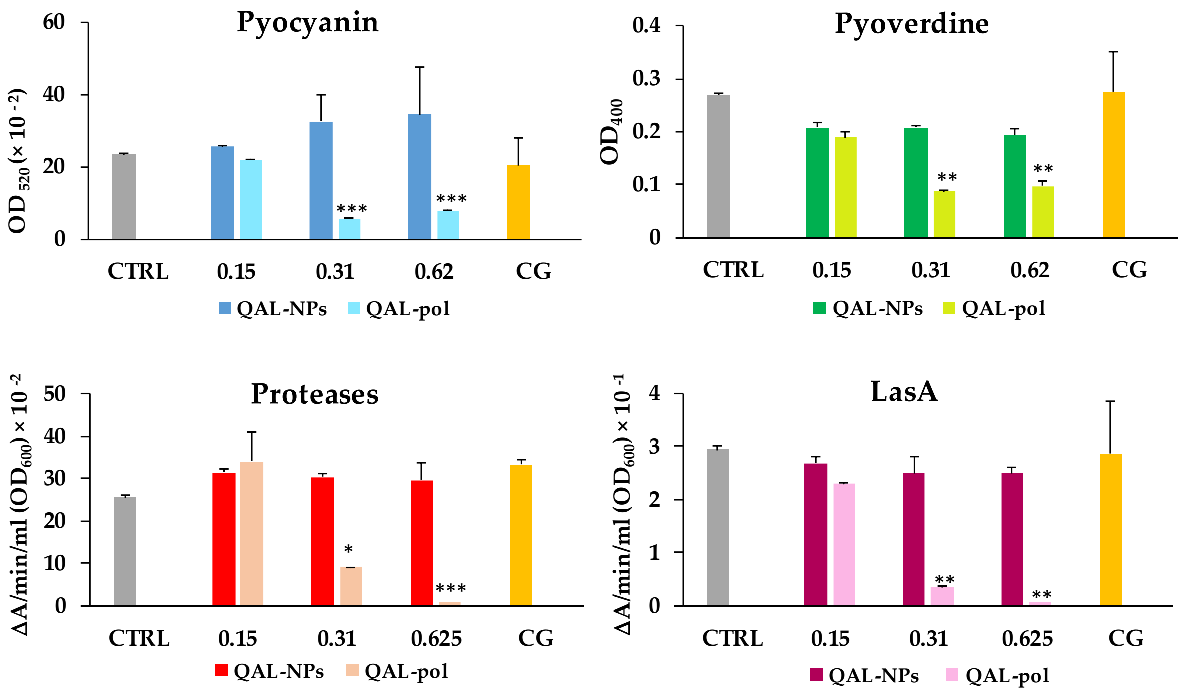

Figure S1: QAL-NPs were assayed for their anti-virulence activity against the Pa W4 strain. To this aim the strain was incubated at 37°C for 48 h in static conditions with concentrations of QAL-NP corresponding to 0.155, 0.31 and 0.62 mg/mL of QAL. CTRL: bacteria without treatment. CG: carrageenan was tested at the concentration of 0.1 mg/mL corresponding to the concentration of CG present in QAL-NPs equivalent at 0.62 mg/mL of QAL. Results are expressed as the means and error standard of the mean from three independent experiments. (*$p<0.05$; **$p<0.01$; ***$p< 0.001$).
